# Comparison of acidifying agents and clotrimazole for treatment of otomycosis: a comprehensive one-way mini-review

**DOI:** 10.18502/cmm.2023.345035.1402

**Published:** 2023-06

**Authors:** Zeynab Yassin, Behrooz Amirzargar, Reza Ghasemi, Farnaz Valizadeh, Mahsa Fattahi

**Affiliations:** 1 Antimicrobial Resistance Research Center, Iran University of Medical Sciences, Tehran, Iran; 2 Otorhinolaryngology Research Center, Department of Otorhinolaryngology-Head and Neck Surgery, Tehran University of Medical Sciences, Tehran, Iran; 3 Student Research Committee, School of Medicine, Shahid Beheshti University of Medical Sciences, Tehran, Iran; 4 Department of Cell and Molecular Biology, Kish International Campus, University of Tehran, Tehran, Iran; 5 Immunology, Asthma and Allergy Research Institute, Tehran University of Medical Sciences, Tehran, Iran; 6 Children’s Medical Center, Pediatric Center of Excellence, Tehran University of Medical Sciences, Tehran, Iran

**Keywords:** Acetic acid, Boric acid, Clotrimazole, Otomycosis

## Abstract

**Background and Purpose:**

This review aimed to compare the efficacy of acidifying agents and clotrimazole in the treatment of patients with otomycosis.

**Materials and Methods:**

The databases, including Research Gate, Google Scholar, ScienceDirect, Embase, Medline, Scopus, Cochrane, and library databases of clinical trials were searched in this study. The keywords were "Fungal Ear Infection" and "Otitis External" for otomycosis, "Clotrimazole", Lotrimin", "Mycelex", "Desenex", and "Clotrimaderm Mycoderm" for clotrimazole, and "Boric Acid Alcohol", "Alcohol-vinegar solution", Burow solution (Domeboro), "Isopropyl Alcohol", "VoSol" and "Acetic Acid" for acidifying agents. Regarding search strategy, a total of 53 studies were collected, 11 of which were maintained for assessment. Almost all studies were published after 1990. These articles discussed the role of clotrimazole and acidifying compounds in the treatment of otomycosis. Moreover, the route of administration, dosage, and side effects of these medications were highlighted.

**Results:**

Eight studies had similar results and claimed that clotrimazole has the best or most significant effect on the treatment of otomycosis for patients suffering from pain, itching, swelling, and hearing loss.

**Conclusion:**

Although all medications appear effective, there is a paucity of evidence to fully support the decision to choose between clotrimazole or acidifying agents for the treatment of otomycosis in terms of both efficacy and safety. However, in the biomedical field, the re-emerging investigation attention is due to the statements of a number of mechanisms defending the use of acidifying agents to treat mycosis (of antifungal-resistant species).

## Introduction

Otomycosis is a superficial fungal infection caused by fungal growth within the external auditory canal of the ear. It is a widespread infection worldwide; however, it is more prevalent in tropical and subtropical regions, including Iran [ 1 ]. Predisposing factors for this disease include manipulation of the ears, humid and hot environment, age, predisposing primary bacterial infection, immune system disorders, swimming, pregnancy, and previous use of broad-spectrum antibiotic or steroid agents [ 2 , 3 ]. Clinical symptoms in most patients are pruritus, occasional otalgia, aural fullness, and hypoacusis [ 4 , 5 ].

Otomycosis accounts for approximately 15-20% of otitis externa and an increase in incidence of its cases has been reported [ 6 ]. The best diagnostic method is a sampling of discharges and finally microscopic observation of fungal elements, including hyphae, pseudohyphae, and yeast cells [ 7 ]. The three main causes of this infection are saprophytic fungi (70%), yeasts (20-25%), and dermatophytes (5%), among which *Aspergillus* spp. and *Candida albicans* are the most common agents [ 8 , 9 ].

The epidemiological profile of otomycosis varies based on various factors in different populations and countries. However, what is clear is that the prevalence of this infection is higher in areas with hot and humid climates and hot seasons. In terms of age, most studies have generally shown that most cases occur among young and middle-aged individuals. The etiological agents are mostly filamentous fungi, the most principal of which is *Aspergillus* species. Among *Aspergillus* spp., *A. niger*, and *A. flavus* are the most common ones [ 1 , 9 ].

Nowadays otomycosis management has created challenges for patients and physicians due to its prolonged course of treatment and probability of relapse. The best treatment for this disease is clearing the auditory canal of the ear and using topical medications [ 10 , 11 ]. Specific topical medications include clotrimazole, miconazole, and nystatin, and non-specific drugs consist of acidifying agents and gentian violet [ 12 ]. Clotrimazole works by damaging the penetrability barrier of the cytoplasmic membrane of the fungi, which produces pits in the cell membrane, and leaking out the substances of the fungi, consequently destroying the fungus and healing the disease.  Following clotrimazole drop therapy, signs expressive of otomycosis do not indicate the risk of the reappearance of the illness and due to its economical pricing and easy convenience, is commonly suggested by otolaryngologists.

Acidifying agents may be used in mild, acute, or chronic cases with minimal otalgia, but their main usage is in the preventative care of patients who are prone to developing recurrent acute or chronic otitis externally. Alkaline pH has been shown to be a risk factor for the development of acute or chronic otitis external with loss of acidity proportional to the degree of otitis external; therefore, restoration of the natural acidity of the external canal can inhibit the growth of bacteria responsible for otitis external. 

The following are commonly used acidifying solutions for mild, chronic, or preventative care of the external canal: boric acid alcohol; alcohol-vinegar solution (alcohol, one-fourth white vinegar, and one-fourth distilled water); acetic acid in aluminum acetate drops (Domebro), also known as Burow solution; isopropyl alcohol; propylene glycol and acetic acid otic solution (Vosol); and acetic acid [ 1 , 10 , 11 ].

**Figure 1:**
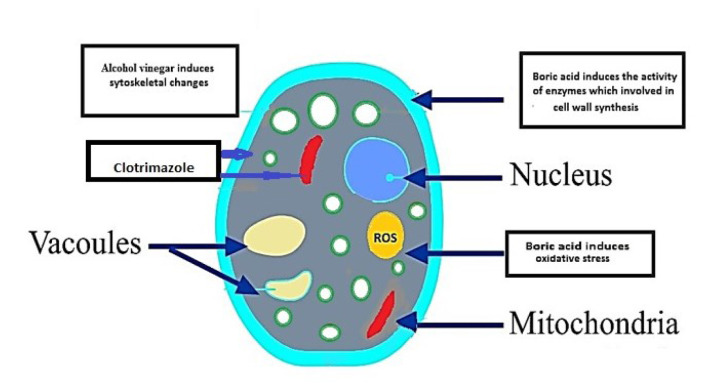
A schematic of the general mechanisms of action of some acidifying agents on fungal cell walls. ROS: Reactive oxygen species in comparison to clotrimazole.

A schematic of the general mechanisms of action of some acidifying agents on fungal cell walls is presented in Figure 1. Boric acid, more exclusively orthoboric acid, is a complex of oxygen, boron, and hydrogen with formula B (OH). It is also known as boracic acid or hydrogen borate and is typically considered brightness crystals or a bleach powder that liquefies in water and appears in nature as the inorganic sassolite. 

Therefore, there are many policies for handling or reducing fungal diseases, such as the increase of obstructions, the inadequacy of nutrients, and the addition of mediators breaking fungal constructions and/or their critical mechanisms [ 13 ]. Presently, boric acid presents a useful means for the treatment of candidiasis [ 14 ]. It can play the role of the blocker of azole-resistant *C. albicans* [ 15 ]. Boric acid has fungistatic to fungicidal effects on *C. albicans*, based on their accumulation and temperature. Blockage of oxidative metabolism seems to be a crucial antifungal process, and interruption of hyphal transformation and biofilm maturity is significant; accordingly, inhibition of virulence may have impacts on curative efficiency *in vivo*.

Alcoholic vinegar has a gentle hydrogen ability resulting in the dissemination of acetic acid. Moreover, its likely interface with the enzymes involved in the development of ergosterol, a main element of fungal plasma membranes, can describe the anti-*Candida* properties of alcoholic vinegar [ 16 , 17 ].

*In vitro* investigations presented a relationship between anti-bacterial properties and the combination of acetic acid and aluminum acetate. In fact, aluminum is a toxic mediator, which has been shown to modify the molecular construction of cell membranes, leading to alterations in its functional properties. 

Despite the global spread, there has been a limited number of studies performed in this regard. Moreover, there is no absolute and definitive treatment for this infection. Therefore, the recurrence of the disease is often observed. As a previous comparative study has been carried out on acidifying agents and clotrimazole for the treatment of otomycosis, this one-way review study aimed to compare the effectiveness and adverse events associated with these antifungal agents.

## Materials and Methods

### Search strategy

An electronic literature search was carried out by the authors in Google Scholar, Embase, Medline, and Scopus. The keywords were "Fungal Ear Infection" and "Otitis External" for otomycosis, "Clotrimazole", Lotrimin", "Mycelex", "Desenex", and "Clotrimaderm Mycoderm" for clotrimazole, and "Boric Acid Alcohol”, “Alcohol-vinegar solution", Burow solution (Domeboro), "Isopropyl Alcohol", "VoSol”, and “Acetic Acid” for acidifying agents. 

The titles and abstracts of the studies were reviewed for the determination of their suitability and eligibility. Retained full-text articles were then subjected to qualitative and quantitative evaluation. At all the steps, any disagreements were resolved through the meeting of the authors. It should be mentioned that only the English articles were included in this study. The full versions of the manuscripts were reviewed in terms of their English writing, and the plagiarism was also assessed. The manuscripts were scientifically revised. Diseases occurring in the presence of another ear or systemic disease were excluded from the review. Moreover, studies in languages other than English were also excluded from the present study.

### Data extraction

The data were collected, and the accuracy of the data was confirmed by researchers. Epidemiologic information was extracted from the selected articles. Furthermore, general information was obtained from them, including publication year, study design, geographical state and country, sample size, number of patients with a proven fungal ear infection, inclusion and exclusion criteria of each study, and average age and gender of the participants. 

Diagnosis and treatment outcomes were also characterized. The information about the type of species and frequency of the most frequent human fungal pathogens were documented. Details of interventions were collected, including treatment concentration, presence of a steroid molecule, mode of administration (i.e., cream, drops, and powder), and dose and number of administrations per day. The primary draft of the article was prepared, and the manuscript was scientifically revised.

## Results

Regarding the search strategy, in total, 53 articles were collected, 11 of which were maintained for assessment. Table 1 summarizes a review of the key information of the included studies. All the studies were published after 1990 and were original studies, clinical trials, or case reports. In five studies, only clotrimazole (cream, ear drop, or lotion) was used as a singular medication for otomycosis cases [ 10 , 18 - 21 ]. There were several reports on the combination of clotrimazole and acidifying compounds [ 22 - 26 ]. It is noteworthy that there was just one study in which acidifying agents were used as monotherapy for otomycosis [ 1 ]. 

**Table 1:** Demographic data, procedure, and treatment of fungal agents in different available studies.

Adverse effects	Pathogen(s)	Steroid	Route of administration	Dosage	Drugs	Examination		Gender	Average age	No. of patients	Design/year	Study	Reference
						Culture-based	Direct microscopy						
Local allergy	*A. niger*	No	Ear Drop	4 drops, every 8 h for 4 weeks	Clotrimazole 1%	+	+	Male: 24 (46.15%)	ND	52	Clinical Trial / 1993	Sheikh et al.	[ 22 ]
	*Candida* spp.		Ear Drop	4 drops, every 12 h for 2 weeks	Acetic acid in alcohol 2%			Female: 28 (53.85%)					
	*Mucor* spp.		Ear Drop	4 drops, every 8 h for 3 weeks	Acetic acid in water 2%								
	*Penicillium* spp.												
Irritation	*A. niger*	No	Ear Drop	3 drops, every 8 h for 2 weeks	Clotrimazole 1%	+	+	Female: 49 (49%)	ND	100	Prospective / 2003	Pradhan et al.	[ 18 ]
	*A. fumigatus*							Male: 51 (51%)					
	*A. flavus*												
	*C. albicans*												
	*Mucor* spp.												
No	*C. albicans*	No	Lotion	4 drops, every 8 h for 4 weeks	Clotrimazole 1%	+	+	Male: 1 (50%)	19	2	Prospective / 2003	Jadhav et al.	[ 19 ]
								Female: 1 (50%)					
ND	*Scedosporiumapiospermum*	Dexamethasone 0.1%	Ear drop	ND	Clotrimazole	+	+	Male: 1	8	1	Case report / 2004	Bhally et al.	[ 20 ]
ND	*C. albicans*	No	Ear Drop	ND	Clotrimazole	+	-	Male: 94 (57%)	4.08	166	Retrospective / 2005	Martin et al.	[ 23 ]
	*C. parapsilosis*		Ear Drop		Acetic acid			Female: 72 (43%)					
	*A. fumigatus*												
ND	ND	B-Hydrocortisone	ND	ND	Clotrimazole	-	+	Male: 75 (56%)	47.6	132	Retrospective / 2006	Ho et al.	[ 10 ]
								Female: 57 (43%)					
ND	*Malassezia* spp.	Hydrocortisone	Powder	1 week	Clotrimazole	+	+	Male: 1	Age: 31	1	Case report / 2010	Latha et al.	[ 24 ]
			Topical solution		Acetic acid 2%								
ND	*Aspergillus* spp.	No	ND	Every 8 h for 3 weeks	90 ml Isopropyl alcohol + 10 ml Acetic acid 2%	+	+	Male: 16 (30.7%)	ND	52	Clinical Trial / 2010	Yeganeh-Moghadam et al.	[ 27 ]
	*C. albicans*							Female: 36 (69.3%)					
	*Mucor* spp.												
	*Rhizopus* spp.												
	*Scopulapriopsis* spp.												
ND	ND	No	Ear Drop	3 drops, every 8 h for 2 weeks	Clotrimazole	-	+	Male to female ratio:1.86	36.65	100	Clinical Trial / 2012	Malik et al.	[ 25 ]
			Ear Drop	3 drops, every 8 h for 2 weeks	Salicylic acid 3%								
burning sensation 5.6% / Irritation 3.7% / Itching 22.8% / other 5.3%	ND	No	Ear drop	1 dose in a week	Clotrimazole 1%	+	+	Male: 46 (38.3%)	41	120	Clinical Trial / 2016	Romsaithong et al.	[ 26 ]
burning sensation 14.8% / Irritation 14.8% / Itching 11.5% / other 6.4%					3% boric acid in 70% alcohol			Female: 74 (61.6%)					
ND	ND	ND	Cream	Single-dose	Clotrimazole 1%	+	+	Male: 22 (55%)	28.6	40	Prospective / 2019	Dundar et al.	[ 21 ]
								Female: 18 (45%)					

According to Table 1, the populations included in these studies were small, ranging from 1 to 166 subjects. There were different inclusion and exclusion criteria associated with comorbidities. Physical examination was performed for diagnosis in all studies. Moreover, molecular procedures were applied in none of these articles. Based on direct microscopic and culture-based examinations, the most frequently obtained fungal agent was *Aspergillus* spp., followed by *Candida* spp., *Mucor* spp., and *Penicillium* spp. (Table 1). 

Due to the insufficient number of direct comparisons of these medications, it was impossible to carry out a direct comparison of the medicines. Therefore, a multiple one-way analysis was conducted using studies performed on clotrimazole and acidifying agents. Only three articles had documented adverse effects of these medications on patients [ 18 , 22 , 26 ] and toxicity was not mentioned in any investigation.

In a report performed by Pradhan and colleagues, the efficacy of clotrimazole 1% was evaluated in 100 patients [ 18 ]. Their presenting symptoms included pain, itching, fullness, and discharge and middle ear effusion. After one month of clotrimazole therapy, all patients recovered, except three of them [ 18 ]. Jadhav et al. stated that two patients recovered from otomycosis after one month of topical administration of clotrimazole 1% [ 19 ]. 

Dundar et al. highlighted that the post-treatment scores of pains and itching were significantly lower than the pre-treatment scores (*P*<0.01) [ 27 ]. In addition, the follow-up examinations demonstrated that 95% of the ear canals were entirely clean and symptoms (perforated and intact tympanic membrane, itching, pain, fullness, hearing loss, tinnitus) were resolved. Besides, at a single visit, they suggested that clotrimazole 1% cream was efficient for the treatment of otomycosis [ 27 ]. 

Results of the study performed by Bhally et al. were consistent with those of previous studies which showed that surgical debridement and topical administration of clotrimazole can be used to manage otomycosis. In their study, otomycosis was due to *Scedosporium apiospermum* in a child presented with debris in the external auditory canal and discharge in the last 1-2 weeks [ 20 ]. In addition, findings of a study conducted by Ho et al. supported those of previous studies in the literature which suggested clotrimazole as a good choice for the treatment of otomycosis patients who exhibited several symptoms and signs, such as otalgia, otorrhea, hearing loss, aural fullness, pruritus, and tinnitus [ 10 ]. 

Results of a study performed by Yeganeh-Moghadam et al. significantly differed from those of the previous research discussed in the literature [ 1 ]. In the aforementioned study, only acidifying compounds were were applied to combat otomycosis. A mixture of isopropyl alcohol and acetic acid 2% was used in patients with a history of itching, inflammation, discharge, and hearing difficulty. Their report demonstrated that 41 (78.8%) cases were treated successfully in three weeks; however, relapse of the disease was recorded in four cases [ 1 ].

In a study performed by Martin et al., all patients who were treated with clotrimazole or acetic acid were cured [ 23 ]. In their study, the mean time of treatment was 3.8 weeks [ 23 ]. Another research was conducted by Sheikh et al. in 1993 in Iran on 52 individuals [ 22 ]. Their clinical findings included debris and mass in the external auditory canal, pain, itching, hearing loss, and discharge. They compared the efficacy of clotrimazole 1%, acetic acid in alcohol 2%, and acetic acid in water 2%. After the period of treatment (4 and 2 weeks for clotrimazole 1% and acetic acid in alcohol 2%, respectively), the cure rate was, 73.68% for clotrimazole 1% and 50% for acetic acid in alcohol 2%. Notably, none of the patients responded to acetic acid in water 2% [ 22 ]. 

Results of another study performed by Romsaithong et al. on the comparison of clotrimazole ear drops and boric acid 70% in otomycosis patients demonstrated that clotrimazole 1% solution is significantly more effective than 3% boric acid in 70% alcohol [ 26 ]. Itching, otalgia, aural fullness, and otorrhea, were recorded in their patients. In that study, the effectiveness of clotrimazole 1% solution in otomycosis treatment was 85.2%; moreover, the efficacy of boric acid was reported as 67.3% [ 26 ]. 

Latha et al. presented a patient with mild pain (on and off), hypoacusis, and mild hearing loss who was diagnosed with otomycosis due to *Malassezia* spp. [ 24 ]. After two weeks of clotrimazole and acetic acid 2% consumption, the patient was cured [ 24 ]. Malik et al. studied the efficacy of clotrimazole in comparison with salicylic acid 3% in patients with itching, blockage, and discharge [ 25 ]. Both medications were administered with equal dosage and duration of consumption. Their study revealed that salicylic acid 3% improved ear itching better than clotrimazole. It also decreased the ear blockade better than clotrimazole. Moreover, regarding the clearance of fungal debris/discharge, clotrimazole had better results [ 25 ]. 

In terms of geographical region, clotrimazole is still a popular drug all around the world, especially in Asia. In the current review, it can be observed that the majority of Asian studies were conducted in South Asian countries, such as India [ 27 , 28 - 30 ], East Asian countries, such as Thailand [ 26 ], and the Middle East countries, such as Iran [ 31 ] and Turkey [ 21 ]. In addition, one article was carried out in Mexico in Central America [ 32 ]. The administration of acidifying agents in recent articles has been reduced; furthermore, many countries are still using these drugs, such as Turkey, and no reason was observed to decrease the use of this medication.

## Conclusion

Although all medications appear effective, there is a paucity of evidence to fully support the decision to choose between clotrimazole or acidifying agents for the treatment of otomycosis in terms of efficacy and safety. However, in the biomedical field, the re-emerging investigation attention is due to the statements of a number of mechanisms defending the use of acidifying agents to treat mycosis (of antifungal-resistant species).
